# LncRNA RMRP knockdown upregulates PD-1 expression in natural killer cells

**DOI:** 10.1038/s41598-025-20720-4

**Published:** 2025-10-03

**Authors:** Sarah Abouhegaziah, Doris Urlaub, Ahmed Moustafa, Maha Mostafa, Amged Ouf, Khaled Abou-Aisha, Carsten Watzl, Mona Rady

**Affiliations:** 1 Microbiology, Immunology and Biotechnology Department, Faculty of Pharmacy and Biotechnology, The German University in Cairo, Cairo, Egypt; 2 Leibniz Research Centre for Working Environment and Human Factors (IfADo), TU Dortmund, Dortmund, Germany; 3 Department of Biology, American University in Cairo, New Cairo, Egypt; 4 Systems Genomics Lab, American University in Cairo, New Cairo, Egypt; 5 Generations GENETICS, Cairo, Egypt; 6 Faculty of Biotechnology, German International University, New Administrative Capital, Cairo, Egypt

**Keywords:** Long non-coding RNA (lncRNA), RMRP, PD-1, Natural killer (NK) cells, Immune checkpoint, Immunotherapy, Gene expression, Cytokine stimulation, Flow cytometry, Gene regulation in immune cells, Immune evasion, Immunogenetics, Innate immune cells, Innate immunity, Lymphocytes, Tumour immunology

## Abstract

The role of long non-coding RNAs (lncRNAs) in Natural Killer (NK) cells is still an ongoing research question. LncRNAs are RNAs with a length of more than 200 nucleotides. While they do not code for functional proteins, they have many regulatory roles from transcriptional to post-translational mechanisms that can affect gene expression. On the other hand, NK cells are large, innate, granular immune cells that exert cytotoxic effects against virus-infected and malignant cells using various mechanisms of actions. In this study, we aimed to perform functional analysis of lncRNAs in NK cells. We focused on five lncRNAs: MALAT1, RMRP, LINC-PINT, GAS5, and LINC00299. Our findings revealed that the expressions of the five lncRNAs were downregulated in cultured NK cells and upon cytokine stimulation. In addition, we observed an upregulation of the PD-1 receptor on the surface of NK cells in response to knocking down RMRP. This suggests that RMRP may have an inhibitory effect on PD-1 expression in NK cells. The proposed inverse relationship between RMRP and PD-1 offers new insights into the regulation of PD-1 expression and its role in NK cell function. Given PD-1’s critical role as an immune checkpoint, by understanding the regulatory relationship between RMRP and PD-1, new strategies can be explored to enhance NK cell efficacy, opening up new possibilities for designing immunotherapies that restore NK cell function by potentially targeting PD-1 regulation.

**Supplementary Information:**

The online version contains supplementary material available at 10.1038/s41598-025-20720-4.

## Introduction

Only 2% of the human genome codes for proteins, while the rest has once been regarded as “junk”, without any known function. Yet, up until recent years, accumulating evidence has proven the importance of non-coding DNA regions. It has been discovered that a fraction of the non-coding DNA encodes for long-noncoding RNAs (lncRNAs), which are RNAs longer than 200 nucleotides that do not code for functional proteins^1^. While lncRNAs can act as epigenetic regulators that can affect gene expression through DNA methylation, histone modification, and gene silencing, they have also been found to regulate mRNA processing by affecting the splicing of pre-mRNA or affecting mRNA editing^2–8^. Furthermore, LncRNAs can regulate post-translational modifications of proteins through hydroxylation, phosphorylation and ubiquitination, which can affect protein activity^3,9,10^. LncRNAs’ expression levels may vary depending on the type of tissue they are localized in and can also be affected by various medical conditions, including cancer^11^. Until recently, more than 45,000 lncRNAs have been discovered in humans, yet most of them still require extensive research to truly understand their functions and mechanism of action. Furthermore, most known functions of lncRNAs to date are associated with either tumor progression or tumor suppression. High levels of GAS5 and LINC-PINT have been found to have a tumor-suppressive action, while on the other hand, elevated levels of MALAT1 and RMRP relay a pro-oncogenic action in various types of cancers^12–15^.

LncRNAs have been linked to various immune cells, including macrophages, T- and B-lymphocytes, but little is known about the role of lncRNAs in NK cells. Comprising around 15% of the total peripheral blood mononuclear cells (PBMCs) and found in various tissues, natural killer cells are large, granular, innate immune cells that recognize and kill virus-infected cells and cancer cells^16^. Arising from common lymphoid progenitor cells, NK cells development, which includes maturation, expansion and acquisition of specific receptors mainly occurs in the bone marrow with the aid of various factors such as transcription factors, cytokines, and growth factors to obtain mature NK cells^17–21^. NK cells can be divided by the presence of CD56 (specific NK cell marker) and CD16 (Fc receptor for IgG) on their surface into ${\text{CD}}56^{{{\text{dim}}}} {\text{CD}}16^{ + }$ and ${\text{CD}}56^{{{\text{bright}}}} {\text{CD}}16^{ - }$^22^. Upon activation and contact with target cells, NK cells kill virus-infected and tumor cells mainly through degranulation, which involves the release of cytotoxic granules that induce apoptosis in target cells^23,24^. NK cells’ functional ability is mediated via a complex assortment of signals arising from their inhibitory and activating receptors. While NKG2A and KIRs are HLA-specific inhibitory receptors that restrict NK cells’ activity upon binding to MHC class I on the surface of target cells, other inhibitory receptors exert their repressive action upon interacting with a wide range of ligands^18,25,26^. Programmed cell death protein 1, or PD-1, is an essential immune checkpoint that is usually described in T-cells but, upon recent years, has been garnering increasing attention in NK cells. PD-1 is an inhibitory NK cell receptor that binds to PD-L1 and PD-L2 to suppress NK cell cytotoxic function. PD-1 is hardly expressed on the surface of resting NK cells. Still, its level of expression increases under certain pathological conditions such as cytomegalovirus (CMV) infections and certain types of cancer, such as Hodgkin lymphoma, Kaposi sarcoma, and ovarian carcinoma^27^.

In this study, we aimed to uncover the gene expression levels and functional roles of RMRP in primary human NK cells using QPCR and ASO Gapmer knockdown technology to enhance our understanding of novel mechanisms regulating NK cell functions. Through flow cytometry, we identified a potential link between RMRP and PD-1, providing new insights into the functional relationship between lncRNAs and NK cell activity.

## Methods

### Cell culture

K562 human myelogenous leukemia cells (ATCC®CCL-243™) were maintained in IMDM medium (Thermo Fisher) with 10% FCS (Thermo Fisher) and 1% penicillin/streptomycin (P/S, Thermo Fisher). The cells were split three times a week to an end concentration of 0.3 × 10^6^ cells/mL. To obtain primary human natural killer cells, PBMCs were isolated from the fresh blood of healthy donors using density gradient centrifugation. Afterwards, primary human natural killer cells were isolated from PBMCs using Dynabeads® Untouched™ Human NK Cells Kit (Thermo Fisher) according to the manufacturer’s instructions. Natural Killer cells were maintained in culture in IMDM GlutaMAX medium (Thermo Fisher) with 10% FCS and 1% penicillin/streptomycin (P/S) by using irradiated K562-mb15-41BBL feeder cells (gift from Dario Campana; St. Jude Children’s Research Hospital, Memphis, TN) (5 × 10^5^ cells/mL) and IL-2 (100 U/ml; NIH Cytokine Repository) for the first 10 days with the addition of IL-21 (100 ng/ml; Miltenyi Biotec) in day 1 following isolation. Subsequently, according to the cell concentration, medium exchange or splitting of cells was performed every 2–3 days with the addition of IL-2 (100 U/ml) and IL-15 (5 ng/ml; Pan-Biotech). The expansion of NK cells took 4 weeks and all experiments were performed using NK cells with a culturing duration of 4–5 weeks. All cells were incubated at 5% (v/v) CO2 at 37 °C.

### Quantitative polymerase chain reaction (QPCR)

To perform Quantitative Polymerase Chain Reaction (QPCR), RNA was first isolated from NK cells using RNeasy Mini Kit (Qiagen) according to the manufacturer’s instructions while using QIAshredder spin columns (Qiagen) for the homogenization of lysates. The isolated RNA’s concentration and integrity was measured using Eppendorf BioSpectrometer® (Eppendorf AG). Afterwards, Reverse transcription was carried out using QuantiTect Reverse Transcription Kit (Qiagen) according to the manufacturer’s instructions on RNA volume equivalent to 200 ng. After performing reverse transcription, the obtained cDNA was used for QPCR using TaqMan Probes (Qiagen) targeting the selected lncRNAs (Supplementary Table 1). Using QuantiNova Probe PCR Kit (Qiagen), QPCR reactions were performed by mixing 10 μL Probe PCR Master Mix, 1 μL selected Taq-man probe, 1 μL template cDNA and 8 μL RNase-free water to a total reaction volume of 20 μL in PCR tubes. QPCR was later carried out in Bio-Rad CFX96 Real-Time System (C1000 Touch Thermocycler) at an initial 2 min at 95 °C then cycled at 5 s at 95 °C and 5 s at 60 °C for 40 cycles. Each sample was run in triplicates and two negative controls were always included: No Reverse Transcriptase (NRT) control and No Template Control (NTC). The obtained CT values were normalized to an internal reference control [Ribosomal Protein large P0 (RPLP0)] and the level of gene expression was calculated using the $2^{ - \Delta \Delta ct}$ method, with control samples set to 1. 1 represents no change in gene expression, less than 1 represents down-regulation and more than 1 represents up-regulation (fold-enrichment).

### Cytokine stimulation assay

Three types of samples were used: non-stimulated sample (without cytokines), IL-2 & IL-15 stimulated sample and IL-12 & IL-18 stimulated sample. For the non-stimulated sample, we used NK cells without the addition of any cytokines while on the other hand, for the IL-2/IL-15 stimulated sample, we used NK cells with the addition of IL-2 (100 U/ml) and IL-15 (5 ng/mL). For the IL-12/IL-18 stimulated sample, we used NK cells with the addition of IL-12 (5 ng/mL; R&D Systems) and IL-18 (25 ng/mL; Biozol). Two million NK cells were used for each type of sample and all samples were suspended in IMDM GlutaMAX medium (Thermo Fisher) with 10% FCS and 1% penicillin/streptomycin (P/S) with a concentration of 2.5 million/mL. All samples were plated at 100 μL per well in round-bottom plates and incubated at 5% (v/v) CO2 at 37 °C overnight with QPCR performed the subsequent day.

### Knockdown of RMRP in NK cells by gymnosis

Previously cultured, two million NK cells were harvested and resuspended in IMDM GlutaMAX medium (Thermo Fisher) with 10% FCS and 1% penicillin/streptomycin (P/S) (700 μL medium/2 million cells) with the addition of IL-2 (100 U/ml) and IL-15 (5 ng/mL). Next, the needed volume of ASO gapmers required to achieve the intended concentrations (500 nM/1 μM/4 μM) was added to each knockdown sample, except the normal sample did not receive any gapmers (Supplementary Table 2). Lastly, the cells were plated at 180 μL/well in round-bottom plates and incubated at 5% (v/v) CO2 at 37 °C for 3 days, after which QPCR was performed to verify the knockdown.

### Flow cytometry analysis of IFN-γ, CD107a & MIP1β

To test the level of IFN-γ, CD107a & MIP1β, we used the three main knockdown samples: normal NK cells (without knockdown), negative control NK cells (with negative control ASO gapmer) and knockdown NK cells (with ASO gapmer targeting the selected lncRNAs). Each of the three samples was divided into two main sub-samples: NK cells only (stained), and NK cells mixed with K562 cells (stained). 0.1 million NK cells were harvested from each sample for each sub-sample, and 0.1 million K562 cells for each of the mixed samples. In V-shaped 96-well plates, all sub-samples were resuspended in 50 µL of K562 medium containing CD107a (APC). Afterwards, the plate was incubated at 5% (v/v) CO2 at 37 °C for 1 h. After 1 h, we added (1:1000) Brefeldin A (Sigma-Aldrich) into each well and incubated again for 2 h. After 2 h, all wells were centrifuged and were resuspended in 25 μL of PBS containing Zombie NIR (Biolegend). The plate was then incubated in the dark at room temperature for 15 min after which 25 μL of FACS buffer (PBS/2% FCS) containing CD56 BV421 (BD Bioscience) was added to each well. Again, the plate was then incubated in the dark at room temperature for 15 min then all wells were washed with 150 μL of PBS and resuspended in 50 μL of FACS buffer and 50 μL of 4% Paraformaldehyde (Sigma-Aldrich). Then, the plate was incubated in the dark at room temperature for 20 min. After incubation, the plate was centrifuged, and the cell pellets were resuspended in 50 μL of BD FACS™ Permeabilizing Solution 2 (BD Bioscience) and incubated in the dark at room temperature for 10 min. Afterwards, all wells were washed with 150 μL of FACS buffer. Then, all sub-samples were resuspended in 25 μL of FACS buffer containing antibodies against IFN-γ (FITC, Biolegend) and MIP1β (PE, BD Bioscience). The plate was then incubated in the dark at room temperature for 20 min after which all wells were washed with 150 μL of FACS buffer. Lastly, all wells were resuspended in 150 μL of FACS buffer containing 2% formaldehyde and stored at 2–8 °C to analyze the samples using BD LSRFortessa™ Cell Analyzer (BD Bioscience) the next day. All data were analyzed using FlowJo (version 10; FlowJo LLC). All antibodies and dilutions are listed in Supplementary Table 3.

### Flow cytometry of NK cell surface receptors

To perform NK cell surface receptor staining, two panels of antibodies were used: An Activator panel in which we tested for most of the activating receptors and a mixed panel in which we tested for a wide range of receptors. A staining master mix was prepared for each panel in which we mixed the needed antibodies with Brilliant Stain Buffer (BD Bioscience) and FACS buffer. 0.15 million NK cells were harvested from each sample for each panel and placed in a 96-well V-shaped plate. All samples were stained with Zombie (NIR) in PBS and incubated in the dark at room temperature for 15 min after which they were washed with FACS buffer and resuspended in 50 μL of each reaction master mix respectively. Again, the plate was incubated in the dark at room temperature for 20 min after which it was washed twice with FACS buffer and resuspended in 150 μL of FACS buffer to be analyzed using Cytek® Aurora (Cytek Bioscience). All data were analyzed using FlowJo (version 10; FlowJo LLC). All antibodies and dilutions are listed in Supplementary Table 3.

### Statistical analysis

Statistical analyses were performed using GraphPad PRISM version 9 (GraphPad Software, Inc). One-way ANOVA with Tukey’s test as a post-hoc test for multiple comparisons was used to evaluate possible differences between samples (*: *P* < 0.05) (**: *P* < 0.01) (***: *P* < 0.001), with *P* > 0.05 being non-significant.

## Results

### Selection of lncRNAs candidates for functional analysis

Based on next-generation sequencing (NGS) data from total RNA isolated from freshly obtained NK cells of healthy individuals (unpublished data generated at our laboratory), we identified the top 20 highly expressed lncRNAs in NK cells (TPM > 100). From this selection, we chose five lncRNAs with previously annotated functions to measure their gene expression levels in primary human NK cells (Supplementary Table 4). These lncRNAs are: (1) Metastasis-associated lung adenocarcinoma transcript 1 (MALAT1), (2) RNA component of mitochondrial RNA processing endoribonuclease (RMRP), (3) Long intergenic non-protein coding RNA, p53-induced noncoding transcript (LINC-PINT), 4) Growth arrest-specific 5 (GAS5), and (5) Long intergenic non-protein coding RNA 299 (LINC00299). The expression levels (TPM) of the full list of lncRNAs in the NK cell sample are provided in Supplementary Table 6.

### Downregulation of RMRP in primary cultured NK cells

**Fig. 1:**
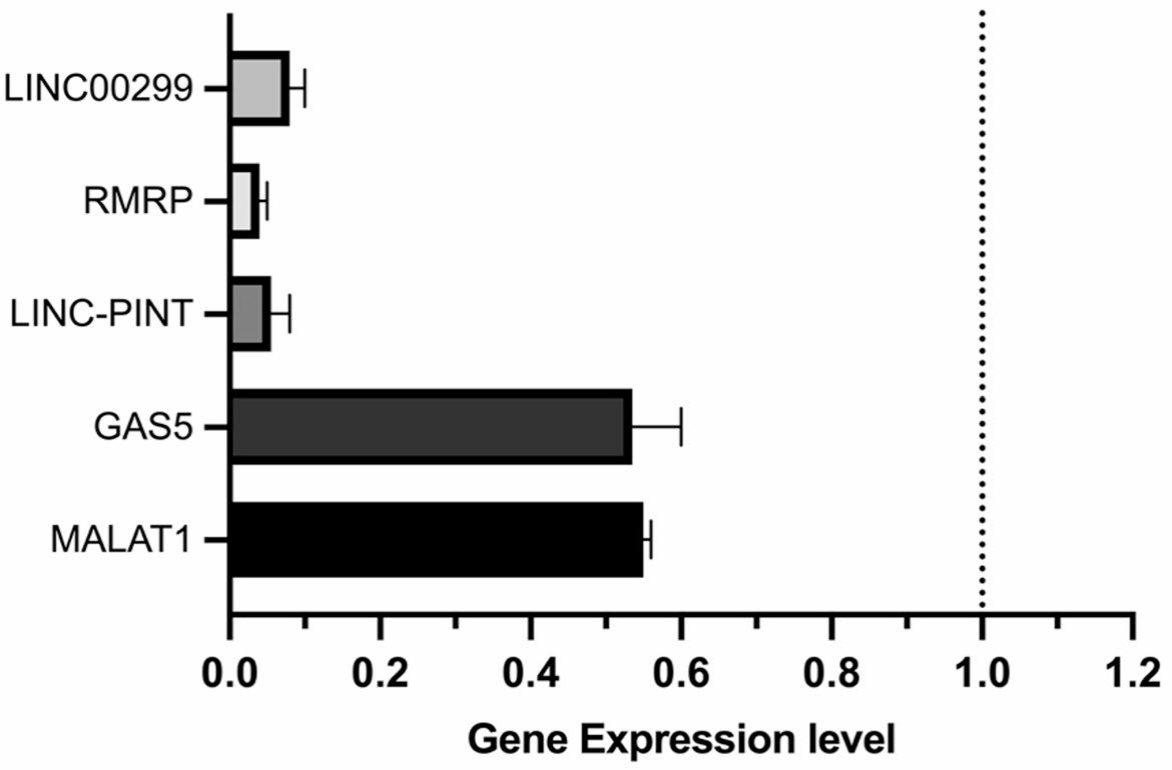
Gene expression levels of MALAT1, GAS5, RMRP, LINC-PINT and LINC00299 in fresh versus cultured NK cells. QPCR to measure lncRNA expression in cultured NK cells samples normalized to fresh NK cells samples shows downregulation of the selected 5 lncRNAs: MALAT1, GAS5, RMRP, LINC-PINT and LINC00299. Dotted line represents our reference (1). Results are shown as mean fold of gene expression ± SEM. (n = 2).

As little is known about the gene expression of the selected lncRNAs in NK cells and to investigate if culturing of NK cells subsequently affects their expression, we isolated total RNA from freshly isolated NK cells and 1-month cultured NK cells from the same two donors and performed QPCR to detect possible differences in expression. The fresh samples served as our control, to which we compared the cultured samples. Figure 1 shows the mean relative gene expression levels of the selected 5 lncRNAs of fresh versus cultured NK cells samples from two donors. As displayed, all genes were downregulated upon culturing. RMRP (25-fold change and *P* = 0.0066), LINC-PINT (18-fold change and *P* = 0.0168), and LINC00299 (12.5-fold change and *P* = 0.0138) showed high levels of downregulation upon culturing, with RMRP showing the highest difference in gene expression.

### Cytokine stimulation downregulated RMRP expression in NK cells

**Fig. 2:**
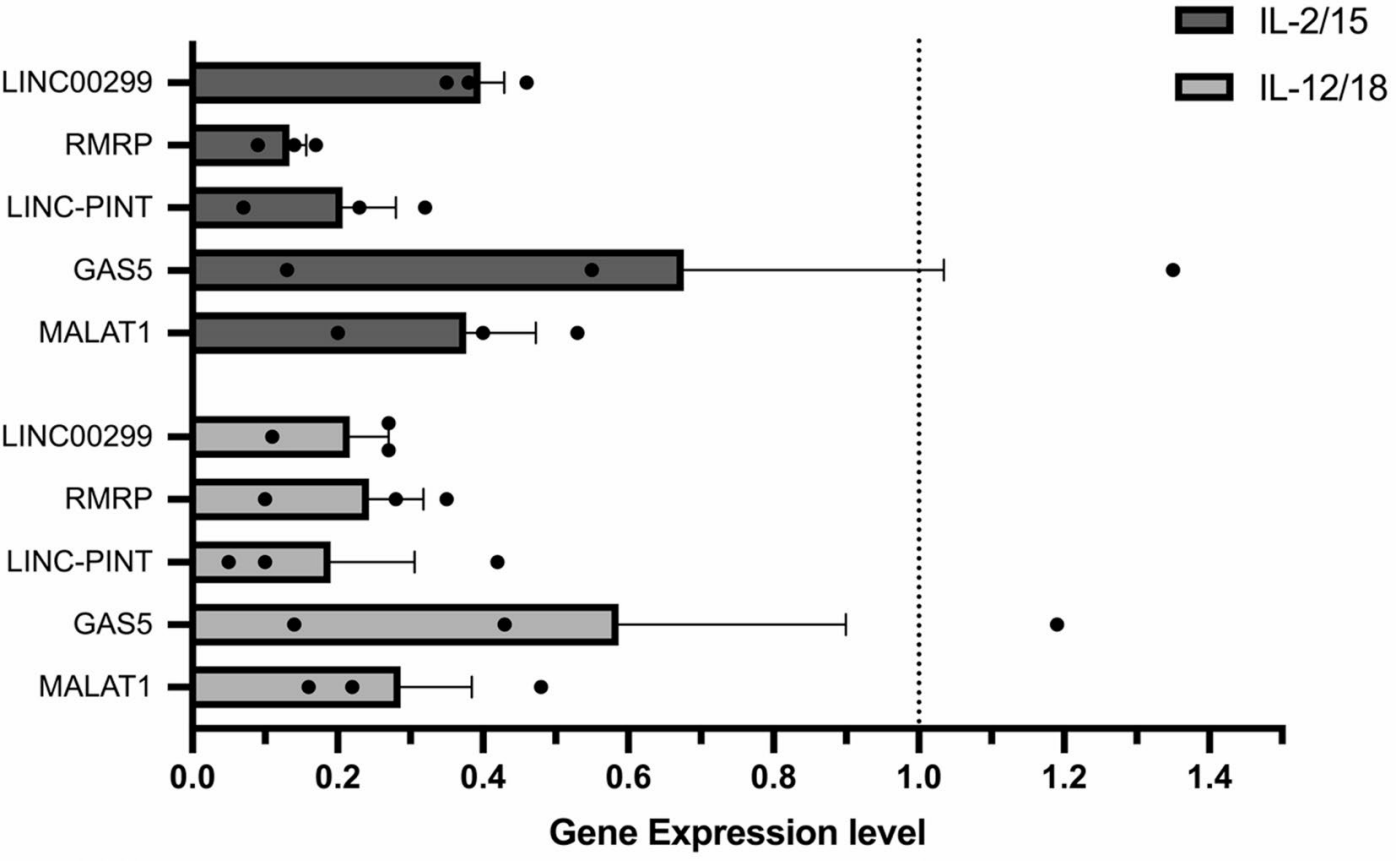
Gene expression levels of MALAT1, GAS5, RMRP, LINC-PINT and LINC00299 in cytokine stimulated NK cells. QPCR of lncRNAs in NK cells samples stimulated with IL-2/IL-15 and samples stimulated with IL-12/IL-18 show varying levels of downregulation of the selected 5 lncRNAs: MALAT1, GAS5, RMRP, LINC-PINT and LINC00299. Dotted line represents our reference (1). Single dots represent our 3 donors. Results are shown as mean fold of gene expression ± SEM, (n = 3).

Furthermore, we wanted to study the possible effect of cytokines on the gene expression MALAT1, GAS5, RMRP, LINC-PINT and LINC00299) using IL-2, IL-15, IL-12 and IL-18 as they are proven as NK cell stimulators. We divided our samples into a non-stimulated sample (without cytokines), IL-2 and IL-15 stimulated sample, and IL-12 and IL-18 stimulated sample. The non-stimulated sample (without cytokines) served as our control to which we compared the IL-2/IL-15 stimulated sample and IL-12/IL-18 stimulated sample for 24 h. As shown in Fig. 2, all the five lncRNAs had a significant decrease in the levels of gene expression in both IL-2/IL-15 stimulated samples and IL-12/IL-18 stimulated samples. MALAT1, GAS5 and LINC00299 had a lower level of gene expression in the IL-12/IL-18 stimulated samples (3.5 (*P* = 0.0184), 1.7 (*P* = 0.3176), and 4.6 (*P* = 0.0046) fold changes, respectively) compared to the IL-2/IL-15 stimulated samples (2.6 (*P* = 0.0229), 1.4 (*P* = 0.4616), and 2.5 (*P* = 0.0029) fold changes, respectively). On the other hand, RMRP had a lower level of gene expression in the IL-2/IL-15 stimulated samples (7.5-fold change and *P* = 0.0007) compared to the IL-12/IL-18 stimulated samples (fourfold change and *P* = 0.0095). LINC-PINT had a slightly lower level of gene expression in the IL-12/IL-18 stimulated samples (5.2-fold change and *P* = 0.0199) compared to the IL-2/IL-15 stimulated samples (fivefold change and *P* = 0.0084). In addition, GAS5 showed an unstable level of gene expression in both IL-2/IL-15 stimulated samples and IL-12/IL-18 stimulated samples with varying levels of expression between up-regulation and down-regulation.

### RMRP knockdown did not affect NK cell functionality

**Fig. 3:**
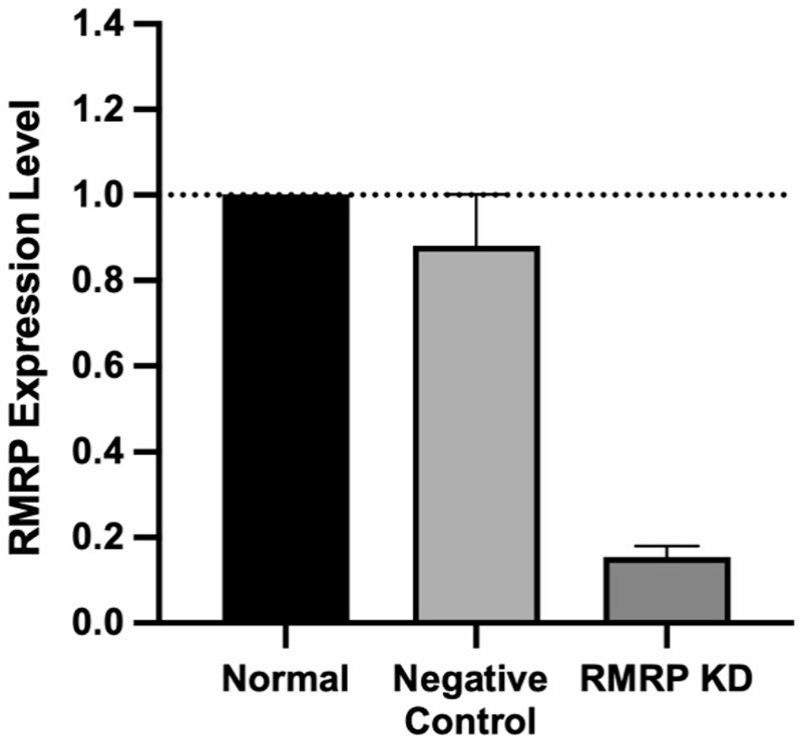
Knockdown of RMRP in NK cells. RMRP expression level in normal NK cells (untransfected; set at 1), negative control (transfected with negative control ASO gapmer), RMRP Knockdown (using 500 nM of ASO Gapmer). Knockdown using unassisted uptake resulted in approximately 80% knockdown. Results are shown as mean fold of gene expression ± SEM. (n = 2).

To investigate the functional role of the different lncRNAs, we performed knock-down experiments in NK cells. Unfortunately, only the knock-down of RMRP was successful (Supplementary Table 5). When using Gymnosis (unassisted uptake), and 500 nM ASO gapmer for an incubation period of 3 days, we observed a knockdown of approximately 80% knockdown (Fig. 3). All knockdown experiments were validated using QPCR. To perform the functional analysis assays, we used the RMRP knockdown NK cells and compared their results to two controls: normal NK cells (without knockdown) and negative control NK cells (with negative control ASO gapmer).

To test RMRP’s functional activity in NK cells, we performed a combined assay in which we tested for degranulation, cytokine release, and chemokine levels. To test for degranulation, we measured the surface expression of lysozyme-associated protein CD107a, a degranulation marker, to quantify the degranulation of cytotoxic granules. Moreover, we measured the level of NK cells’ cytokine release through interferon-γ (IFN-γ), a stimulatory cytokine. Lastly, to determine the chemokine levels secreted by NK cells, we measured the level of Macrophage Inflammatory Protein-1 beta (MIP1β), which can induce the activation of various granulocytes and stimulate the production of other pro-inflammatory cytokines.

There were no substantial differences in the levels of CD107a, IFN-γ, and MIP1β for the three K562-stimulated NK cell samples: normal (untransfected) NK cells, negative control NK cells, and RMRP knockdown NK cells. (Supplementary Fig. 1).

### RMRP knockdown increased PD-1 expression in NK cells

We performed NK cell surface receptor staining, or phenotypic staining, in which we stained for a lot of the NK cells’ surface receptors, including activating and deactivating receptors using fluorophore-labelled antibodies, and compared their levels between RMRP knockdown NK cells, normal NK cells, and negative control NK cells. To measure the level of the fluorophore-labelled antibodies, we used Cytek® Aurora (Cytek Bioscience), which can distinguish up to 40 fluorophores at the same time, consequently allowing the determination of all the stained receptors simultaneously. Two panels were used to test for all the target receptors as shown in Table 1.

**Table 1:** NK cell surface receptor staining panels.

Activator panel	Mixed panel
CD3	CD38
CD56	NKG2C
NKP46	CD3
2B4	KLRB1
NKP44	CD56
CD16	4-1BB
DNAM-1	KLRG1
NKG2D	HLA-DR
NKP30	FasL
Zombie NIR	TIGIT
	CD11a
	CD8
	CD27
	NKG2A
	CTLA-4
	TRAIL
	PD-1
	CD18
	TIM-3
	Zombie NIR

Table showcasing panels used for NK cell surface receptor staining. Activator panel stains for most of the activating receptors, while Mixed panel stains for a wide range of receptors, including activating and deactivating and adhesion receptors.

**Fig. 4:**
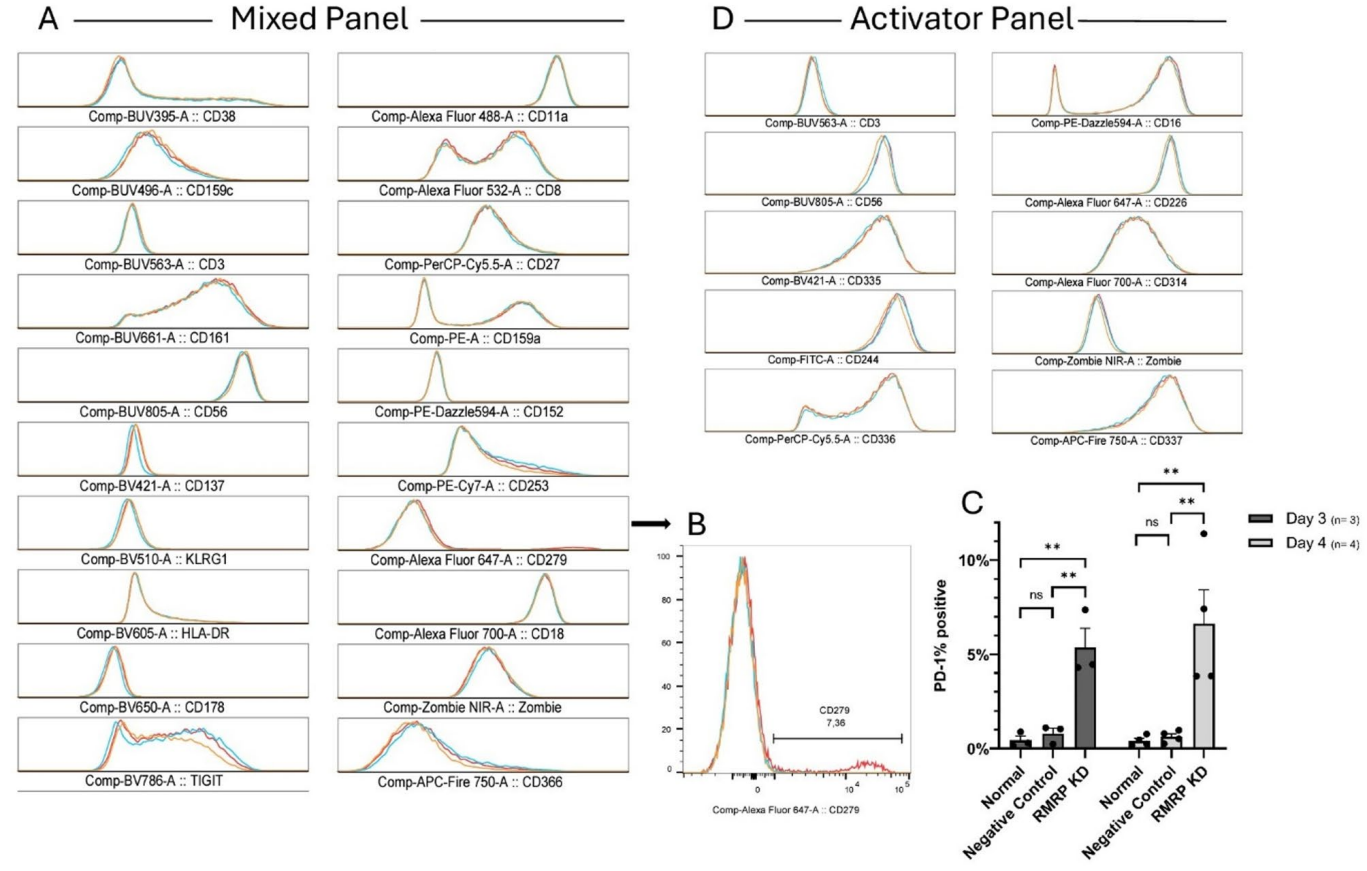
NK Cell Surface Receptor Staining. (**A**) Histogram overlay of the Mixed panel for the three NK cell samples: normal NK cells (orange), negative control NK cells (blue), RMRP Knockdown NK cells (red). (**B**) Overlay of the three samples to showcase the PD-1% positive increase in RMRP knockdown NK cells (red) in comparison to our two controls. (**C**) Bar-diagram showcasing that PD-1% is significantly higher in RMRP knockdown NK cells in comparison to both Normal and negative control NK cells following both incubation periods (3 days and 4 days). Single dots represent individual donor data. Results are displayed as mean fold of gene expression ± SEM. Differences were analyzed using one-way ANOVA with Tukey’s test as a post-hoc test. [(*: *P* < 0.05) (**: *P* < 0.01) (***: *P* < 0.001). **D)** Histogram overlay of the Activator panel for the three NK cell samples. All Overlays were generated using FlowJo.

As illustrated in Fig. 4A,D, there was no considerable difference in the levels of all surface receptors in both panels between RMRP knockdown NK cells and normal NK cells and negative control NK cells except in the level of the deactivating surface receptor Programmed Death-1 (PD-1; CD279); the PD-1 level was higher in RMRP knockdown NK cells in comparison to normal NK cells and negative control NK cells. PD-1 is a surface receptor that when bound to its ligands PD-L1/ PD-L2, results in the deactivation of the immune cells’ cytotoxic ability. PD-1 is a known immune checkpoint since inhibiting it will lift the restraint on immune cells’ ability to attack viral cells and cancer cells. Subsequently, we repeated our assay multiple times using four different donors and comparing the level of PD-1 after 3 days and after 4 days to check if the level of PD-1 will remain higher in RMRP knockdown NK cells in comparison to our controls and if the level will change between donors and following different incubation periods. As demonstrated in Fig. 4B,C, there was a significant difference (*P* < 0.01) in the level of PD-1 between RMRP knockdown NK cells and both our controls (normal NK cells and negative control NK cells) following our two incubation periods (3 days and 4 days) in all our 4 donors. Based on our obtained results, we can preliminarily conclude that RMRP has an inhibitory effect on the expression of PD-1 in NK cells since its knockdown results in an increase in the level of PD-1 in comparison to our controls. This means that we can hypothesize that increasing the level of RMRP may aid NK cells in their activation and cytotoxic ability by downregulating PD-1.

## Discussion

Over the years, long non-coding RNAs (LncRNAs) have been garnering increased interest as a noteworthy topic of research. Various research projects have been dedicated to uncovering lncRNAs’ functional role in various types of cells, including cancer cells and, more recently, immune cells. Many lncRNAs’ functions have been linked to various immune cells, including macrophages, and T-cells yet little to almost nothing is known about lncRNAs’ functions in natural killer (NK) cells. This study aimed to reveal the impact of MALAT1, GAS5, RMRP, LINC-PINT, and LINC00299 lncRNAs on NK cell functions. Surprisingly, all five selected lncRNAs were downregulated upon culturing, with RMRP showing the highest level of downregulation. This obtained result raises the question of whether these lncRNAs’ play a role in NK cells. In addition, MALAT1, RMRP, LINC-PINT, and LINC00299 were downregulated upon IL-2/IL-15 and IL-12/IL-18 stimulation, this result increased the curiosity behind understanding the exact mechanism behind this downregulation upon NK cell stimulation. On the other hand, as GAS5 has been previously found to have a stimulatory effect on NK cells as its overexpression results in increased IFN-γ production and cytotoxicity^28^, its varying level of gene expression between upregulation and downregulation upon NK cell stimulation requires further studies to truly understand the connection between its level of expression and the NK cell functionality.

After testing for IFN-γ, CD107a, and MIP1β in the absence of RMRP, the lack of variation in the level of IFN-γ, CD107a, and MIP1β between control NK cell samples and RMRP knockdown NK cells raised the question of whether RMRP truly plays a role in NK cells or not. Consequently, NK cell surface receptor staining was used to investigate the role of RMRP in a bigger picture by testing to find if there is a link between RMRP and any of the NK cell receptors. Remarkably, PD-1 levels were up-regulated following RMRP knockdown in NK cells compared to control samples.

RMRP, the RNA component of mitochondrial RNA processing endoribonuclease, is a dual-localized lncRNA expressed in both the nucleus and the cytoplasm. RMRP has been claimed to have a pro-oncogenic role in many types of cancer cells. Still, in addition to its cancer promoting role, RMRP has been found to regulate RNA processing in both mitochondria and ribosomes^15^. However, until recently, the role of RMRP has not been fully investigated in NK cells, and no link has been established between RMRP and any functional or regulatory mechanisms in NK cells. The current project has proven that RMRP is indeed expressed in NK cells and is downregulated upon culturing and cytokine stimulation. Yet, more in-depth studies are required to truly establish the gene expression of RMRP in NK cells and determine, precisely, the mechanism behind its downregulation. In an attempt at uncovering potential links between RMRP and NK cells, we have found that RMRP knockdown results in the up-regulation of PD-1 on the surface of NK cells, which opens a new door for further research as PD-1 is a valuable immune checkpoint that has been extensively studied but has been mainly linked to T-cells.

PD-1 is an inhibitory receptor that, more recently, has been studied on the surface of NK cells. It has been discovered that circulating NK cells in healthy individuals does not express PD-1 and as we hypothesize that RMRP and PD-1 have an inverse relation, we can expect that circulating NK cells in healthy individuals have high levels of RMRP, explaining the absence of PD-1 expression^29^. This hypothesis can be further supported by our result that RMRP is downregulated in cultured NK cells in comparison to freshly isolated NK cells, which indicates that RMRP levels are high in freshly isolated NK cells and might be the reason why PD-1 is not expressed on circulating NK cells. Yet, although circulating NK cells do not express PD-1 on their surface, it has been found that circulating NK cells contain PD-1 mRNA and protein localized in the cytoplasm, which suggests that for RMRP to decrease the level of PD-1, it may have to play a role in the surface expression of PD-1^30^. However, further studies are required to understand the mechanism behind the hypothesized inverse relationship between RMRP and PD-1. In addition, studies performed on the induction of PD-1 on the surface of NK cells found that glucocorticoids, together with IL-12, IL-15, and IL-18 increase the expression of PD-1^31^. This further proves our hypothesis of the inverse relationship between RMRP and PD-1 by supporting our results that RMRP is downregulated upon stimulation with IL-2/IL-15 and IL-12/IL-18, which may increase PD-1 levels.

Since in this study PD-1 was only measured at the cell-surface level using flow cytometry, studying the total expression of PD-1 at the mRNA and protein levels after RMRP knockdown would give further insight into the exact pathway, which could have caused the increase in PD-1 surface expression.

## Limitations of the current study

Most experiments were performed using a small sample size, yet we have taken great care to ensure the quality and consistency of the existing data, and while the current sample size is limited, the observed trends were reproducible across the donors included. Moreover, we believe that our findings, while preliminary, provide a valuable foundation for future studies involving larger cohorts.

In conclusion, further studies must be performed to truly uncover the mechanism of action behind the downregulation of RMRP in NK cells upon culturing and cytokine stimulation and to better understand and confirm the hypothesized inverse relation between RMRP and PD-1. As PD-1 is a valuable immune checkpoint, better understanding its specific mechanism of action and expression in NK cells can help utilize PD-1 as a potential target for immunotherapies to regain NK cells’ functional ability.

## Supplementary Information

Below is the link to the electronic supplementary material.

Supplementary Material 1

Supplementary Material 2

## Data Availability

The gene expression data from RNA-seq datasets generated and/or analyzed during the current study are available in the supplementary files associated with this manuscript.
