# Supplementary material for: LncRNA RMRP knockdown upregulates PD-1 expression in natural killer cells
Source: Sci Rep. 2025 Oct 3;15:34473. doi: 10.1038/s41598-025-20720-4 (PMC12494946; doi:10.1038/s41598-025-20720-4)
Supplement: Supplementary file 1 — Supplementary Material 1 [file 41598_2025_20720_MOESM1_ESM.docx]

# Supplementary Materials

Supplementary Table 1: TaqMan Probes targeting the selected lncRNAs used for QPCR.

| lncRNA Name | Taq-Man Probe Name | Catalogue Number | Supplier |
| --- | --- | --- | --- |
| LINC00299 | HS_LINC00299_395688 QuantiNova LNA Probe PCR Assay (200) (FAM) | UPFH0274625 | Qiagen |
| RMRP | HS_RMRP_2500009  QuantiNova LNA Probe PCR Assay (200) (FAM) | UPFH1154974 |  |
| MALAT1 | HS_MALAT1_2476235  QuantiNova LNA Probe PCR Assay (200) (FAM) | UPFH1132945 |  |
| GAS5 | HS_GAS5_301749  QuantiNova LNA Probe PCR Assay (200) (FAM) | UPFH0180692 |  |
| RPLP0 | HS_RPLP0_370073  QuantiNova LNA Probe PCR Assay (200) (FAM) | UPFH0249010 |  |
| LINC-PINT | HS_LINC-PINT_610805  QuantiNova LNA Probe PCR Assay (200) (FAM) | UPFH0489724 |  |

Supplementary Table 2: ASO Gapmers targeting the selected lncRNAs for knockdown.

| lncRNA Name | ASO Gapmer Name | Catalogue Number | Supplier |
| --- | --- | --- | --- |
| MALAT1 | MALAT1_1 Antisense LNA™ GapmeR (5 nmol) | 339517 | Qiagen |
| LINC-PINT | LINC-PINT(9T)1 Antisense LNA™ GapmeR  (5 nmol/15 nmol) | 339517/339518 |  |
| RMRP | NR_003051.3_1 Antisense LNA™ GapmeR (5 nmol) | 339517 |  |
| LINC00299 | NR_152741.1_1  Antisense LNA™ GapmeR (5 nmol) | 339517 |  |
| Negative Control | Negative control B Antisense LNA™ GapmeR Control  (5 nmol/15 nmol) | 339515/339516 |  |

Supplementary Table 3: All antibodies and dilutions used for flow cytometry.

| Antibodies | Conjugate | Dilution | Supplier |
| --- | --- | --- | --- |
| CD38 (HB7) | BUV395 | 1:100 | BD Bioscience |
| NKG2C (134591) | BUV496 | 1:50 | BD Bioscience |
| CD3 (UCHT1) | BUV563 | 1:200 | BD Bioscience |
| CD161 (HP-3G10) | BUV661 | 1:100 | BD Bioscience |
| CD56 (B159) | BUV805 | 1:500 | BD Bioscience |
| 41BB (4B4-1) | BV421 | 1:50 | BioLegend |
| KLRG1 (2F1) | BV510 | 1:50 | BioLegend |
| HLA-DR (L243) | BV605 | 1:50 | BioLegend |
| FasL (NOK-1) | BV650 | 1:100 | BD Bioscience |
| TIGIT (741182) | BV786 | 1:100 | BD Bioscience |
| CD11a (HI111) | AF488 | 1:800 | BioLegend |
| CD8a (RPA-T8) | AF532 | 1:200 | Thermo Fischer Scientific |
| CD27 (M-T271) | PerCP-Cy5.5 | 1:50 | BD Bioscience |
| NKG2A (Z199) | PE | 1:100 | Beckman Coulter |
| CTLA-4 (BNI3) | PE-Dazzle | 1:100 | BioLegend |
| TRAIL (N2B2) | PE-Cy7 | 1:50 | BioLegend |
| PD-1 (EH12.1) | AF647 | 1:100 | BD Bioscience |
| CD18 (TS1/18) | AF700 | 1:800 | BioLegend |
| TIM-3 (F38-2E2) | APC-Fire 750 | 1:50 | BioLegend |
| NKp46 (9E2) | BV421 | 1:50 | BioLegend |
| 2B4 (C1.7) | FITC | 1:200 | BioLegend |
| NKp44 (P44-8) | PerCP-Cy5.5 | 1:100 | BioLegend |
| CD16 (3G8) | PE-Dazzle | 1:100 | BioLegend |
| DNAM-1 (DX11) | AF647 | 1:100 | BD Bioscience |
| NKG2D (FAB139N) | AF700 | 1:100 | R & D Systems |
| NKp30 (P30-15) | APC-Fire750 | 1:100 | BioLegend |
| CD56 (NCAM16.2) | BV421 | 1:100 /  1:50 | BD Bioscience |
| CD56 (MEM-188) | APC | 1:100 | BioLegend |
| CD16 (3G8) | FITC | 1:50 | BioLegend |
| CD16 (3G8) | PE | 1:400 | BioLegend |
| CD107a (H4A3) | APC | 1:200 | BioLegend |
| IFN-γ (4S.B3) | FITC | 1:20 | BioLegend |
| MIP1β (D21-1351) | PE | 1:100 | BD Bioscience |

Supplementary Table 4: Top 20 lncRNAs expressed in freshly isolated NK cells with TPMs > 100. TPM: Transcript per million

| ensembl_gene_id | hgnc_symbol | description | transcript_biotype | NKN1 |
| --- | --- | --- | --- | --- |
| ENSG00000251562 | MALAT1 | metastasis associated lung adenocarcinoma transcript 1 [Source:HGNC Symbol;Acc:HGNC:29665] | lncRNA | 14694.3 |
| ENSG00000269900 | RMRP | RNA component of mitochondrial RNA processing endoribonuclease [Source:HGNC Symbol;Acc:HGNC:10031] | lncRNA | 6328.22 |
| ENSG00000227195 | MIR663AHG | MIR663A host gene [Source:HGNC Symbol;Acc:HGNC:27662] | lncRNA | 844.589 |
| ENSG00000259001 |  | ribonuclease P RNA component H1 | lncRNA | 774.86 |
| ENSG00000280800 |  | novel transcript, similar to YY1 associated myogenesis RNA 1 YAM1 | lncRNA | 690.335 |
| ENSG00000274265 |  | novel transcript | lncRNA | 212.249 |
| ENSG00000229807 | XIST | X inactive specific transcript [Source:HGNC Symbol;Acc:HGNC:12810] | lncRNA | 174.282 |
| ENSG00000272917 |  | novel transcript | lncRNA | 153.984 |
| ENSG00000251301 | LINC02384 | long intergenic non-protein coding RNA 2384 [Source:HGNC Symbol;Acc:HGNC:53308] | lncRNA | 152.898 |
| ENSG00000231721 | LINC-PINT | long intergenic non-protein coding RNA, p53 induced transcript [Source:HGNC Symbol;Acc:HGNC:26885] | lncRNA | 149.729 |
| ENSG00000276216 |  | novel transcript | lncRNA | 145.686 |
| ENSG00000257027 |  | novel transcript | lncRNA | 144.123 |
| ENSG00000234741 | GAS5 | growth arrest specific 5 [Source:HGNC Symbol;Acc:HGNC:16355] | lncRNA | 132.34 |
| ENSG00000267322 | SNHG22 | small nucleolar RNA host gene 22 [Source:HGNC Symbol;Acc:HGNC:50285] | lncRNA | 131.795 |
| ENSG00000236790 | LINC00299 | long intergenic non-protein coding RNA 299 [Source:HGNC Symbol;Acc:HGNC:27940] | lncRNA | 128.305 |
| ENSG00000175061 | SNHG29 | small nucleolar RNA host gene 29 [Source:HGNC Symbol;Acc:HGNC:28619] | lncRNA | 120.815 |
| ENSG00000272888 | CHASERR | CHD2 adjacent suppressive regulatory RNA [Source:HGNC Symbol;Acc:HGNC:48626] | lncRNA | 116.022 |
| ENSG00000262202 |  | novel transcript | lncRNA | 114.369 |
| ENSG00000230590 | FTX | FTX transcript, XIST regulator [Source:HGNC Symbol;Acc:HGNC:37190] | lncRNA | 109.095 |
| ENSG00000254911 | SCARNA9 | small Cajal body-specific RNA 9 [Source:HGNC Symbol;Acc:HGNC:32566] | lncRNA | 101.283 |

Supplementary Table 5: Percentage knockdown of the selected lncRNAs using nucleofection and Gymnosis (unassisted uptake). All results are obtained by validating the knockdown using QPCR and normalizing the data to the negative control gapmer NK cell sample. MALAT1 and LINC00299 knockdown using both nucleofection and unassisted uptake proved not successful, while RMRP and LINC-PINT resulted in varying levels of knockdown.

|  | **Concentrations Used** | | | | |  |
| --- | --- | --- | --- | --- | --- | --- |
|  | **Nucleofection** | | **Unassisted Uptake** | | |  |
| **Genes** | 5nM | 25nM | 500nM | 1μM | 4μM |  |
| MALAT1 | 0% | 0% | 0% | 0% | 0% | **% Knockdown** |
| RMRP | N/A | 0% | ~80% | N/A | N/A |  |
| LINC-PINT | N/A | 0% | 0% | ~15% | ~37% |  |
| LINC00299 | N/A | N/A | 0% | 0% | 0% |  |

Supplementary Figure 1: IFN-γ, CD107a & MIP1β Assay on RMRP Knockdown NK cells.


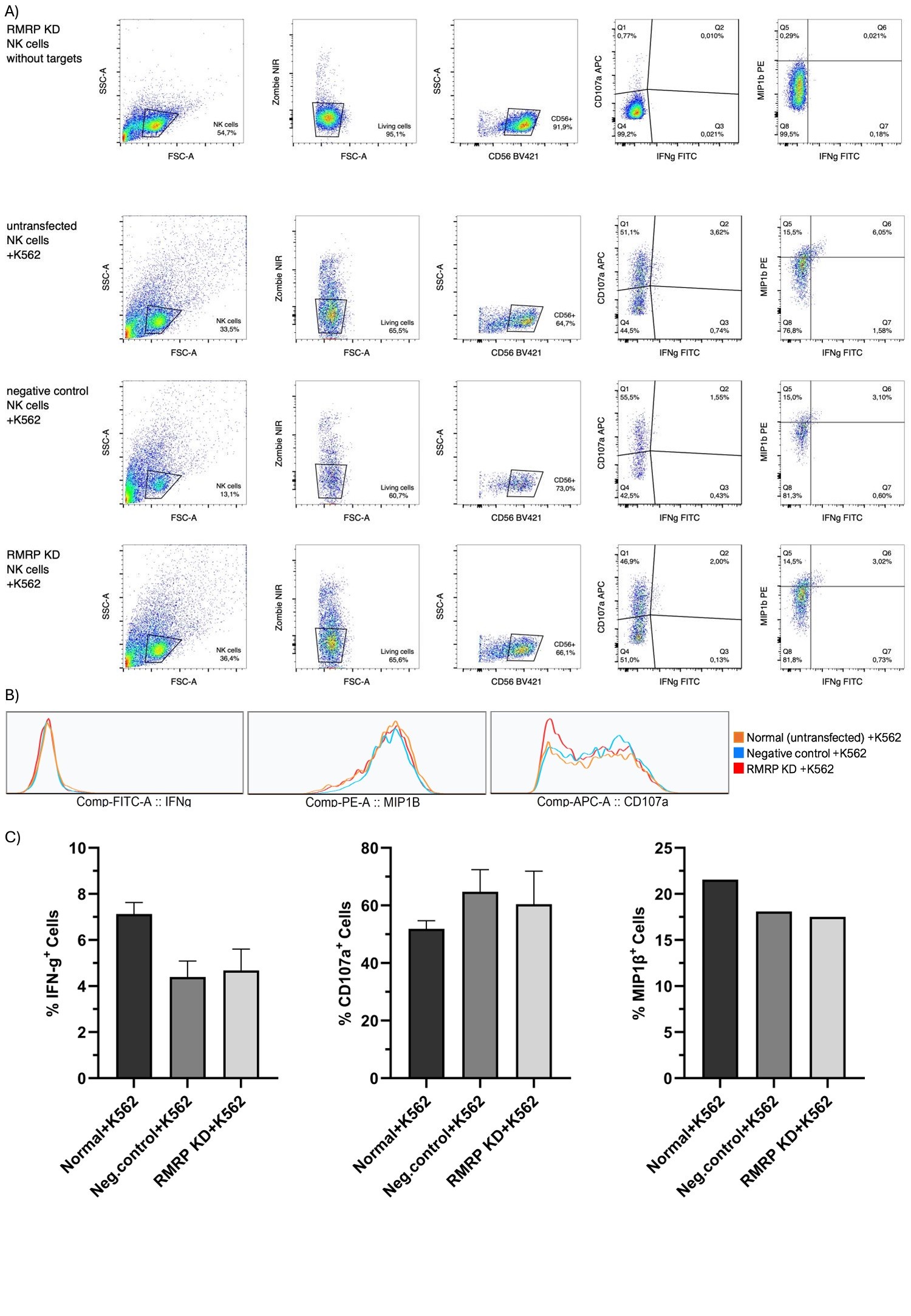


Supplementary Figure 1: IFN-γ, CD107a & MIP1β Assay on RMRP Knockdown NK cells. A) Gating plots for the three K562-stimulated NK cell samples: normal (untransfected) NK cells, negative control NK cells, and RMRP knockdown NK cells. RMRP KD NK cells without targets were used to showcase the changes observed in our three test samples. Pseudo-color plots were generated using FlowJo. B) Histogram overlay of IFN-γ, CD107a, and MIP1β levels for the three K562-stimulated NK samples. Overlay generated using FlowJo. To assess the degranulation, cytokine release and chemokine levels, we stimulated NK cells using K562 myelogenous leukemia cells and then stained CD107a, IFN-γ and MIP1β using fluorophore-labelled antibodies: CD107a (APC), IFN-γ (FITC) and MIP1β (PE). In addition, we used two additional stains: Zombie dye (NIR) which can be used to distinguish between living and dead cells, and CD56 (BV421) which can be used to stain CD56, a NK cell surface marker. We used BD LSRFortessa™ Cell Analyzer (BD Bioscience) to measure the levels of CD107a, IFN-γ, and MIP1β fluorophore-labelled antibodies. C) Bar graphs showcasing the complied data from all donors for each molecule tested: IFN-γ, CD107a, (n=2) and MIP1β (n=1). Results are shown as mean ± SEM.
